# Phonological Codes Constrain Output of Orthographic Codes via Sublexical and Lexical Routes in Chinese Written Production

**DOI:** 10.1371/journal.pone.0124470

**Published:** 2015-04-16

**Authors:** Cheng Wang, Qingfang Zhang

**Affiliations:** 1 Key Laboratory of Behavioral Science, Institute of Psychology, Chinese Academy of Sciences, Beijing, China; 2 Department of Psychology, Renmin University of China, Beijing, China; University College London, UNITED KINGDOM

## Abstract

To what extent do phonological codes constrain orthographic output in handwritten production? We investigated how phonological codes constrain the selection of orthographic codes via sublexical and lexical routes in Chinese written production. Participants wrote down picture names in a picture-naming task in Experiment 1or response words in a symbol—word associative writing task in Experiment 2. A sublexical phonological property of picture names (phonetic regularity: regular vs. irregular) in Experiment 1and a lexical phonological property of response words (homophone density: dense vs. sparse) in Experiment 2, as well as word frequency of the targets in both experiments, were manipulated. A facilitatory effect of word frequency was found in both experiments, in which words with high frequency were produced faster than those with low frequency. More importantly, we observed an inhibitory phonetic regularity effect, in which low-frequency picture names with regular first characters were slower to write than those with irregular ones, and an inhibitory homophone density effect, in which characters with dense homophone density were produced more slowly than those with sparse homophone density. Results suggested that phonological codes constrained handwritten production via lexical and sublexical routes.

## Introduction

Over the past a few decades, many studies have been conducted to investigate the processes and mechanisms underlying spoken production [1–3]. However, investigations devoted to an understanding of written production are limited. In the present study, we investigated how phonological codes constrained the selection of orthographic codes by sublexical and lexical routes in Chinese written production.

A central theoretical issue in the field concerns the extent at which written production is autonomous from or dependent on spoken production. Early theoretical accounts claimed that retrieval of an orthographic representation is entirely dependent on prior retrieval of phonological codes, which is called *obligatory phonological mediation hypothesis*. Evidence supporting this view is derived from the common introspective experience of written production accompanied with inner speech [4], the phonologically mediated spelling errors, such as homophone substitutions (e.g., *there* for *their*) and quasi-homophone substitutions (e.g., *dirth* for *dearth*) [5]. Neuropsychological patients with writing disorders also present comparable impairments in spoken and written language production [6, 7].

However, other neuropsychological studies have demonstrated dissociations between spoken and written production. For example, Rapp, Benzing, and Caramazza [8] presented the case of a neurologically impaired individual who was often able to write the names of pictures correctly, but unable to provide the correct spoken names. Miceli et al. [9] reported a patient who, when presented with a picture, sometimes generated different spoken and written responses (e.g., for a picture of *pliers*, the patient would say “*pincers*” but write *saw*) (see [10] for a similar case study). Some agraphic patients have also been reported to produce errors with phonologically illegal spelling (e.g., [11]). These findings motivated the “*orthographic autonomy hypothesis*,” which assumes that individuals can gain access to orthographic representation directly from meaning without phonological mediation [12]. It should be noted that this account does not necessarily imply that intact writing is unaffected by phonological codes in normal individuals. Several empirical studies recently addressed the role of phonology in orthographic access with chronometric tasks, and have demonstrated that phonological codes indeed influenced writing processes (e.g., [13–21], but see [22, 23]).

**Fig 1:**
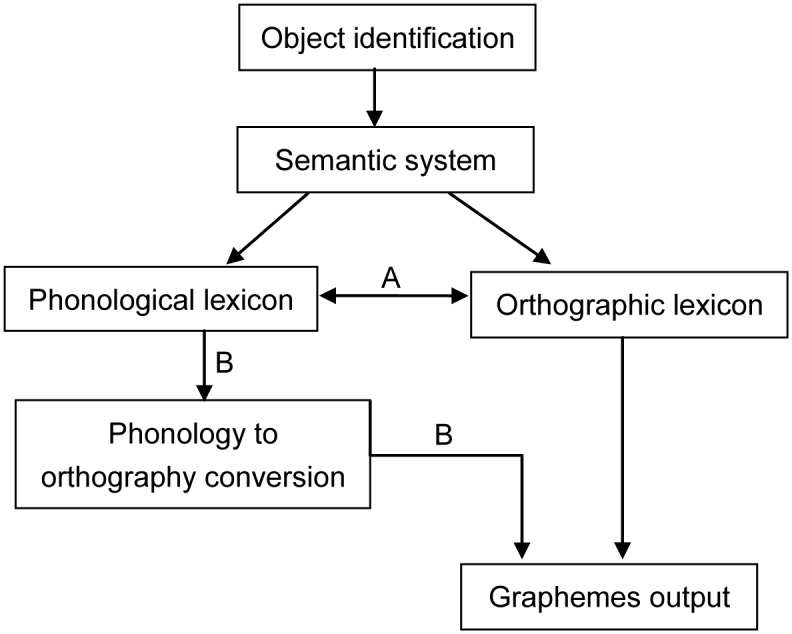
A sketch model of written production

Bonin et al. [14] proposed a written production model (see Fig 1), which assumes a semantic system that is symmetrically linked to both phonological and orthographic output lexicons. This model assumes two routes from phonological lexicon to grapheme output in written production. According to the *lexical* route, phonological and orthographic lexicons directly map onto each other (route A in Fig 1), implying that the selection of graphemic entry is influenced by both direct activation from the semantic system and indirect activation from the phonological lexicon. The sublexical route assumes graphemes are incrementally assembled from phonology via a phoneme-to-grapheme conversion (route B in Fig 1), through which paralleling the sublexical grapheme-to-phoneme conversion route in the dual-route models of reading aloud [24]. Bonin et al. [14] manipulated the consistency of phonology-to-orthography mappings in picture names to identify the potential effects of phonological codes in written picture naming. They found that word-initial inconsistencies at the sublexical level affect writing latencies: Picture names with inconsistent phonology to orthography mapping (e.g., *knife*) were written more slowly than those with consistent ones (e.g., *nose*). Whereas no difference was found when consistency was manipulated at the lexical level (whether picture names had heterographic homophones). In addition to supporting the involvement of phonology in written production, results suggested that phonology affected orthographic encoding mainly via the sublexical route.

However, findings on the role of phonology in written production were inconsistent either. For example, Shen et al. [22] found that additional phonological overlap does not benefit written production in an implicit priming task, which suggested that phonology may not play a role in writing (see also [23]). One possibility is that phonological activation is not mandatory in written production, and it may depend on task demands [16] and target languages in writing [13, 22]. For example, Afonso and Álvarez [13] obtained a phonological overlap effect in Spanish, but Shen et al. [22] did not find this effect in English. This inconsistency might be caused by the fact that Spanish is more transparent than English in sound-to-spelling correspondence. Although some tentative evidence has suggested that phonological codes constrain orthographic output tasks, such as handwriting, more research is needed to resolve this controversial issue.

Evidence for phonological constraints on writing, as mentioned above, comes largely from studies conducted with alphabetic scripts. Findings of phonological activation are not surprising in the case of alphabetic scripts, in which phonological and orthographic codes are closely interrelated. The relationships between phonology and orthography are quasi-systematic in these writing systems. Given that orthography and phonology are largely dissociated in non-alphabetic scripts, such as Chinese, orthographic and phonological effects can be appropriately separated from each other in such scripts by appropriate manipulation. Hence, in non-alphabetic scripts, it is less obvious why orthographic retrieval should be affected by phonological codes. With a picture—word interference (PWI) task, Qu et al. [25] manipulated picture-distractor relationship (distracters were orthographically plus phonologically related, phonologically related, or unrelated to picture names) and stimulus onset asynchrony between onsets of distractors and pictures (SOA: 0, 100, and 200 ms). Priming effects were found for the orthographically plus phonologically related distractors relative to unrelated distractors at 0 and 100 ms SOA, whereas priming from the phonologically related distractors was restricted to 0 ms SOA. These findings suggested that phonological codes are activated rapidly and constrain orthographic output, even in non-alphabetic script (see also [26, 27]).

Although previous studies have found that phonological constraints on Chinese written production [25–27], none of them investigated how different phonological information at the lexical and sublexical routes influence the selection of orthographic codes. Bonin et al. [14] manipulated sound-to-spelling consistency of French words to be written at the lexical (whether target words had heterographic homophones) and sublexical (whether the sound-to-spelling consistency conformed to phoneme-to- grapheme correspondence rules) levels in their study. Following their experimental design, we manipulated phonological properties at the sublexical (route B in Fig 1) and lexical levels (route A in Fig 1) to taps into the two routes in written production in experiments 1 and 2, respectively.

In experiment 1, the phonetic regularity was manipulated at the sublexical level to tap into the process from phonology to orthography conversion (route B). Phonetic regularity in Chinese concerns whether or not a character shares the same syllable and (or) tone with its phonetic radical. In Chinese, the majority (84%) of characters are compound phonograms [28], which consist of a semantic component to indicate the meaning and a phonetic component to indicate the pronunciation. Some phonetic radicals and semantic radicals are characters when they stand alone, so they bear meanings and pronunciations of their own. For example, the character 妈 (pronounced as pinyin /ma1/, meaning *mum*; The pinyin of Chinese character is the official phonetic system for transcribing the Mandarin pronunciation of characters into Latin alphabet; the number following the alphabets indicates characters’ tone.) consists of a semantic radical女(/nv3/, *female*) and phonetic radical马 (/ma3/, *horse*). However, only 38% of characters have phonetic radicals that can indicate their pronunciation [28]. Studies in reading [29–32] have found that the regular characters are reading faster than the irregular ones in Chinese and alphabetical languages, and phonetic regularity effect is modulated by word frequency (WF), i.e., low-frequency words present a significantly larger effect than high-frequency words. Similar regularity effects were also observed in writing to dictation and copying tasks [14, 33–35].

We manipulated phonetic regularity (regular vs. irregular) and WF (high vs. low) in experiment 1, while the lexical properties were matched among conditions. According to the dual-route model in written production [14, 33], the orthographic codes of regular and irregular characters would be generated via the lexical route, and they would through similar processes at the lexical level. The only difference between phonetic regulars and irregulars would originate from the process via the sublexical route. If the sublexical route is involved, we expect a difference on written latencies between phonetic regular and irregular target names. If the sublexical route is not involved, no significant difference would be found in written production.

In experiment 2, the homophone density (HD) at the lexical level was manipulated to tap into the process from phonological lexicon to orthographic lexicon (route A). The HD (or the type measure of syllable frequency) refers to the number of a Chinese character's homophone mates. Studies have demonstrated that HD or syllable frequency modulates linguistic tasks, such as word naming [36–38], spoken picture naming [39, 40], written picture naming [27], and lexical decision [41–45]. The properties at the sublexical level were matched between dense and sparse HD conditions, indicating that the sublexical processes for both conditions would be similar. If phonological and orthographic lexicons are connected through lexical route, written latencies should be longer for dense HD than for sparse HD, becuase there are more homophoone mates competitions for dense HD than for sparse HD.

Additionally, the WF was also manipulated in both experiments. According to the framework proposed by Bonin et al. [14] and findings on word recognition tasks, we predict a WF effect, in which high-frequency words would be produced faster than low-frequency words. The WF was crossed with phonetic regularity in experiment 1 and HD in experiment 2, allowing us to test for any interaction.

## Ethical Statement

The current study was approved by the Independent Ethics Committee of the Institute of Psychology, Chinese Academy of Sciences in Beijing. Written consent was obtained from participants before the administration of the experiments.

## Experiment 1: Regularity Effect

### Methods

#### Participants

Twenty students (7 males; average age: 22.1 years; age range: 20–25 years) participated and were paid a small fee. They were randomly selected from Beijing Forest University and China Agricultural University. All were native Mandarin Chinese speakers with normal or corrected-to-normal vision.

#### Materials

Fifty-six black-and-white line drawings of common objects were selected from Zhang and Yang [46]’s picture database. All had disyllabic names. Half of the picture names were high-frequency words (average: 27.38 per million; range: 12–69 per million), and half were low-frequency words (average: 3.94 per million; range: 0.8–6 per million). Among the high- and low-frequency disyllabic words, half of the first characters were regular, whereas the other half were irregular. Therefore, 56 pictures were divided into four groups of 14 items in two dimensions (i.e., WF and phonetic regularity). The phonetic radicals of regular characters have the same pronunciations as the whole characters [e.g., 轮 (/lun2/, *wheel*), its phonetic radical is仑 (/lun2/, *logical sequence*)], whereas the phonetic radicals of irregular characters cannot provide clues to the whole character’s pronunciation [e.g., character蜡 (/la4/, *wax*) and its phonetic radical昔 (/xi1/, *the past*)]. Among 14 characters in each condition, 13 of them were left—right structured with the phonetic radicals on the right, and one was top-bottom structured with the phonetic radicals at the bottom. Thus, for all target characters, the phonetic components were the second radicals in the writing sequence. Table 1 presents the properties of stimuli, including the frequency and stroke number of the words, first and second characters, none of which were significantly different between regular and irregular conditions (see table 1 for details).

**Table 1:** Mean properties of the picture names used in Experiment 1.

Properties	Low WF			High WF		
	Reg	Irreg	*p*	Reg	Irreg	*p*
Frequency^*a*^ of the words	4.39	3.50	ns	27.69	27.07	ns
Stroke number of the words	18.53	17.60	ns	18.67	17.87	ns
Frequency^*b*^ of the first characters	108	104	ns	118	144	ns
Stroke number of the first characters	11.27	10.00	ns	11.4	10.13	ns
Frequency^*b*^ of the second characters	1186	1065	ns	1255	998	ns
Stroke number of the second characters	7.27	7.60	ns	7.27	7.73	ns
HD of the first characters	6.13	3.67	0.07	5.33	3.73	0.103
Consistency of the first characters	0.71	0.30	0.001	0.75	0.27	<0.001

*Note*: ns = nonsignificant; WF = word frequency; Reg = regular; Irreg = irregular.

^*a*^ Occurrence per million from *Dictionary of Usage Frequency of Modern Chinese Words* [47].

^*b*^ Occurrence per million from *Modern Chinese Frequency Dictionary* [48].

We considered two potential confounding factors, namely, consistency and HD of target characters. In Chinese, some characters consist of the same phonetic radical (this set of characters is called “*phonetic neighbors*” hereafter), and consistency refers to the extent to which these phonetic neighbors are pronounced identically. For example, five characters bear the same phonetic radical啬 (/se4/, *miserly*): 墙(/qiang2/, wall), 蔷 (/qiang2/, a type of rose), 嫱 (/qiang2/, a royal lady), 樯 (/qiang2/, mast), and 穑 (/se4/, to farm). The consistency is 0.8 (4/5) for the former four characters because of identical pinyin among them, but it is 0.2 (1/5) for the last one. Studies on reading Chinese showed that characters with high consistency are read faster than those with low consistency [29, 49]. The other confounding variable considered was HD, which has been proven to modulate various linguistic tasks [27, 36–45].

Matching consistency and HD was difficult when we manipulated WF and regularity and matched frequency and stroke number of words and characters at the same time. Independent *t*-tests showed that regular characters were more consistent than irregular ones in both high-frequency (*t*(26) = 3.76, *p* <.001) and low-frequency conditions (*t*(26) = 5.82, *p* <.001) (see table 1). The HD was marginally different between regular and irregular conditions in the low-frequency words (*t*(26) = 1.85, *p* = .070), and comparable in the high-frequency words (*t*(26) = 1.69, *p* = .103) (see Table 1). Thus, we considered them as covariates in statistical analysis.

#### Design

The experimental design included WF (high vs. low) and phonetic regularity (regular vs. irregular) as within-subjects and between-items factors. Each participant was asked to write 56 target names three times, resulting in a total of 168 trials. Each repetition was set in one block for a total of three blocks. The order of target words within a block was pseudo-randomized to prevent targets with the same onset or radical repetition on consecutive trials. A new sequence was generated for each participant and block.

#### Apparatus

The experiment was conducted using a programmed script with Visual Basic Language. Written latencies (the time intervals between picture onset and initial contact of the pen on the writing surface) were recorded using an IntuosA4 graphic tablet and inking digitizer pen (WACOM, Japan).

#### Procedure

Participants were tested individually. They sat in a quiet room at a comfortable viewing distance in front of the computer. Participants were first asked to familiarize themselves with the experimental stimuli by viewing each picture for 2500 ms with the picture’s name printed below each image.

During the experiment, each trial involved the following sequence. A fixation point (+) was presented at the middle bottom of the screen for 500 ms, followed by a blank screen for 500 ms. A picture was then presented and remained on the screen until the subjects began to write on the tablet. The next trial began 500 ms after the experimenter saw the participant complete the response and pressed a number key that scored the correctness of the responses.

The pictures were displayed at the bottom of the screen to reduce participants’ head and eye movements as they wrote the picture names. During the experiment, participants were instructed to hover the stylus just above the corresponding line on the sheet in anticipation of the response, so that the response would not require arm movement. They were asked to initiate writing picture names as accurately and quickly as possible. Each block began with eight warm-up trials, and breaks were provided between blocks. The whole experiment took approximately 65 min.

### Results

We used the *lmer* program of the lme4 package for estimated fixed effects and parameter estimation of the linear mixed-effect model (LMM) [50, 51]. The free software R [52] was used. Data were analyzed using an LMM that included fixed-effect factors of WF and phonetic regularity, as well as by-participant and by-item random intercepts. Models were fit to the data using a restricted maximum likelihood estimation, which seeks to find parameter values that make the model’s predicted values most similar to the observed values. Model fitting was conducted by initially specifying a model that only included the random factors (participants and items). The HD and consistency were used as covariates in the analysis, which was then enriched by subsequently adding phonetic regularity, WF, and the interaction between WF and phonetic regularity. The best fitting model was defined to be the most complex model that significantly improved the fit over the previous model.

Data from incorrect responses (2.23%), writing latencies longer than 2500 ms or shorter than 300 ms, and latencies deviating 2.5 standard deviations from the cell mean (2.95%) were removed from latency analyses. Table 2 presents the mean of writing latencies and percentage of errors in Experiment 1 by WF and phonetic regularity.

**Table 2:** Mean writing latencies (in ms) and error rates (%) with standard deviation in parentheses as a function of Word Frequency (WF) and Phonetic Regularity in Experiment 1.

WF	Latency		Error rate	
	Irregular	Regular	Irregular	Regular
High	1204 (289)	1163 (263)	2.02 (2.48)	1.31 (1.63)
Low	1178 (274)	1244 (310)	1.55 (2.08)	1.79 (2.17)

Adding factors of phonetic regularity, *χ* ^2^(1) = 0.18, *p* = .671, and WF, *χ* ^2^(1) = 1.02, *p* = .312 did not significantly improve the fit, but adding the interaction between WF and phonetic regularity significantly improve the fit, *χ* ^2^(1) = 6.03, *p* = .014. The best-fitting model thus included WF, *t* = −1.10, *p* = .239, phonetic regularity, *t* = −1.91, *p* = .041, and the interaction between WF and phonetic regularity, *t* = 2.43, *p* = .012). Planned comparison showed that words with the first irregular characters were produced significantly faster than those with regular ones in the low-frequency condition, *t* = 2.23, *p* = .024, but the phonetic regularity effect was not significant in the high-frequency condition, *t* = −1.32, *p* = .158. Furthermore, we added the two controlling factors (HD and consistency) into the best-fitting model to examine whether interactions between WF and phonetic regularity are sustained when HD and consistency are partialed out. However, adding HD, χ2(1) = 1.17, p = .279, or consistency, χ2(1) = 2.49, p = .115, did not significantly improve the fit. In the model including WF, phonetic regularity, the interaction between WF and phonetic regularity, and HD and consistency, the effects of HD, t = −1.41, p = .129, and consistency, t = 1.52, p = .101, were not significant, whereas the interaction between WF and phonetic regularity remained significant, t = 2.43, p = .012.

A parallel analysis was conducted on the errors, but a binomial family was used because of the binary nature of the responses. The best-fitting model included no fixed-effect factors. Adding WF, phonetic regularity, or the interaction between them did not significantly improve the fit. *χ* ^2^(1)s < 0.23, *p*s > .63. Moreover, adding HD and/or consistency did not significantly improve the fit, *χ* ^2^(2) < 0.11, *p*s > .94.

### Discussion

For the writing latencies, a significant interaction between WF and phonetic regularity was observed. The phonetically regular characters were written more slowly than those with phonetically irregular radicals in the low-frequency condition, whereas no effect was observed in the high-frequency condition. These findings indicated that the regularity of sublexical sound-print mapping influenced handwritten production of Chinese characters. Given that most regularity effects found in reading [30, 32, 53, 54] and writing [33–35] are facilitative, the reversed regularity effect and its implications will be discussed in the General Discussion section.

## Experiment 2: Homophone Density Effect

Bonin et al. [14] manipulated the HD (2 vs. 1) of target names to investigate whether a lexical route is present from phonology to orthography in writing French words. Nevertheless, they did not find a significant difference between the two HD groups, thereby confirming the absence of a lexical route in French. However, this conclusion was limited because of the small differences in HD (i.e., 1 vs. 2) between their two experimental conditions. The abundance of homophone mates in Chinese characters enabled us to construct large HD differences between dense and sparse HD conditions (e.g., 10.5 vs. 3.08 for the stimuli in the present experiment, see the materials section). Following Bonin et al.’s experimental logic, we expected HD effects, which would provide evidence for the lexical route involved in writing Chinese. WF was also manipulated because the HD effect is typically more pronounced in the low-frequency words than that in the high-frequency words [38, 41, 44].

In the literature, two standard methods can be used to measure syllable frequency in alphabetic languages, namely, type and token measure of syllable frequency. The first measure is the number of syllabic neighbors, and we refer to it as HD in the present study. The second measure is to calculate the cumulative print frequency of all syllabic neighbors. Both measures affect the linguistic process in various tasks (word naming [36–38], spoken picture naming [39, 40], written picture naming [27], and lexical decision [41–45]). Meanwhile, Conrad et al. [43] found a dissociation of syllable effect between the type and token measures, suggesting that different processes might be involved in the effects of syllable frequency. Thus, in the present study the token syllable frequency was matched between the sparse and dense HD conditions. Other properties at the sublexical level such as phonetic regularity and consistency were also matched.

When the stimuli were matched on properties such as stroke number, token syllable frequency, and consistency and varied on HD and WF, finding sufficient pictures as targets was difficult. Therefore, we adopted a symbol—word associative generation task, which is widely used in speech production domain [39, 40, 55] and written production domain [22]. In this task, participants were asked to memorize four arbitrary pairing relationships between a symbol as a cue and word as a response in the learning phase. The participants then had to generate the corresponding response word upon seeing the cue symbol in the test phase. This task uses words (or characters) rather than pictures as target stimuli, allowing us to find sufficient target words. In general, four pairs were used in symbol-word associative generation task, which is not overloading an adult’s working memory span (see [40]).

Experiment 2 was therefore designed to look for the effects of factors of WF and HD on written production latency. The two factors were factorially crossed in experiment 2, and this allows us to determine whether the two variables have additive or interactive effects according to Sternberg’s “additive factors logic” [56] (see also [40, 57, 58].

### Methods

#### Participants

Twenty college students (10 males; average age: 21.8 years; age range: 19–26 years) from the same student pool participated in the experiment and were paid approximately $10. None of them participated in experiment 1.

#### Materials

Eighty Chinese monosyllabic words were selected as the response words. They were divided into four groups along two dimensions: WF (high vs. low) and HD (dense vs. sparse). Mean lexical frequency for high and low WF conditions were 181.5 (range: 101.2–294.8) and 8.79 (range: 6.1–13.3) per million, respectively. The mean HDs for dense and sparse HD conditions were 11.05 (range: 9–13) and 3.08 (range: 2–4), respectively. Properties of the stimuli, such as stroke number, consistency, and token frequency of the target characters, were statistically matched among the four groups (see Table 3). Furthermore, the proportions of regular characters, irregular characters, and characters that are not phonetic compounds in each condition were also matched between dense and sparse HD conditions in the low, *χ* ^2^(2) = 1.23, *p* = 0.54, and high WF conditions, *χ* ^2^(2) = 0, *p* = 1.

**Table 3:** Mean properties of the target characters used in Experiment 2.

Properties	Low WF			High WF		
	DHD	SHD	*p*	DHD	SHD	*p*
HD^*a*^	3.0	11.2	<0.001	3.2	10.9	<0.001
Word frequency^*b*^	8.1	9.1	ns	179.5	183.6	ns
Token frequency^*c*^	957	1004	ns	987	1057	ns
Stroke number	10.2	10.3	ns	9.7	9.8	ns
Consistency	0.52	0.66	ns	0.45	0.31	ns

*Note*: ns = nonsignificant; WF = word frequency; HD = homophone density; DHD = dense HD; SHD = sparse HD.

^*a*^ Number of homophone mates, from *Modern Chinese Frequency Dictionary* [48].

^*b*^ Occurrence per million from *Modern Chinese Frequency Dictionary* [48].

^*c*^ Cumulative occurrence of homophone mates per million from *Modern Chinese Frequency Dictionary* [48].

Five sets of four symbols consisting of a string of six non-alphabetical signs (e.g., %%%%%% or &&&&&&) were constructed, and they were matched for gross characteristics.

#### Design

The experimental design included WF (high vs. low) and HD (dense vs. sparse) as within-subjects and between-items factors. The experiment consisted of 20 blocks of four symbol—word pairs. Each set was used four times. Within each block, there were four symbol-word pairs, and the four words were selected from each group of the response word, their assignment to four symbols was rotated across participants. The order of blocks was pseudo-randomized with the constraint that the same symbol set was not presented consecutively. Participants were asked to generate each response word four times in each block. The order of symbols in each block was pseudo-randomized with the constraint that the same symbol was not presented consecutively. Each participant completed all blocks, and received a total of 320 experimental trials.

#### Apparatus

The experiment was performed using E-Prime Professional Software (Version 1.1; Psychology Software Tools). Participants were seated in a quiet room approximately 70 cm from a 19-inch monitor. Other aspects were identical to Experiment 1.

#### Procedure

Participants were tested individually. Each block consisted of a learning phase, a practice phase, and a test phase. In the learning phase, four symbol—word pairs were presented on the screen, and participants were asked to memorize them. They were then shown one symbol and asked to write down a response word. All symbols appeared three times in a random order. The participants were asked to repeat the learning and practice phases if they made any wrong responses. After the participants could correctly respond to all 12 symbols, the test phase began.

Each trial involved the following sequence. A fixation point (+) was presented in the middle bottom of the screen for 500 ms, followed by a blank screen for 500 ms. The symbol was presented and remained on the screen until the participant began to write on the tablet. The next trial began 500 ms after the experimenter saw the participant complete the response and pressed a number key that scored the correctness of the responses. Participants were asked to write the response words as accurately and quickly as possible. Breaks were provided between blocks. The 20 experiment blocks took about 140 min in total.

### Results

Data from incorrect responses or technique problem (2.08%), latencies longer than 2500 ms or shorter than 300 ms, and latencies deviating 2.5 standard deviations from the cell means (3.16%) were excluded. Table 4 presents the mean writing latencies and percentage of errors by WF and HD.

**Table 4:** Mean writing latencies (in ms) and error rates (%) with standard deviation in parentheses as a function of Word Frequency (WF) and homophone density (HD) in Experiment 2.

WF	Latency		Error rate	
	Dense HD	Sparse HD	Dense HD	Sparse HD
High	903 (213)	876 (183)	1.00 (1.12)	0.44 (0.73)
Low	953 (241)	934 (190)	1.13 (1.34)	0.56 (1.18)

The data were analyzed using an LMM that included fixed effects of WF and HD, as well as by-participant and by-item random intercepts. Adding HD, *χ* ^2^(1) = 5.66, *p* = .017, and WF, *χ* ^2^(1) = 34.78, *p* = .004, significantly improve the fit, but adding the interaction between WF and HD did not significantly improve the fit, *χ* ^2^(1) = 0.09, *p* = .763. The best-fitting model thus included WF, *t* = 6.52, *p* < .001, and HD, *t* = 2.98, *p* < .01.

A parallel analysis was conducted on the error rates, but a binomial family was used. The best-fitting model included only HD, and error rates for dense HD characters were marginally significantly larger than those for sparse HD characters, Wald *z* = 1.74, *p* = .083. Adding WF, *χ* ^2^(1) = 0.28, *p* = .595, and the interaction between WF and HD, *χ* ^2^(1) = 0.03, *p* = .868, did not significantly improve the fit.

### Discussion

Results replicated the WF effect with a symbol-word associative generation task, which was in line with findings on speech production (e.g., [59]). More importantly, we found that words with dense HD characters took a longer time to write than those with sparse HD ones. According to Sternberg's additive factors logic [54], the absence of the interaction between WF and HD indicated that WF and HD may influence different processing stage of written production, and the HD effect was independent of the WF effect.

For a Chinese character, HD is a phonological characteristic at the lexical level [38]. Thus, the HD effect reflected that phonological codes constrained orthographic outputs in writing via the lexical route from phonology to orthography. The inhibitory HD effect might stem from strong competition from homophone mates in the dense HD condition relative to that in the sparse HD condition.

## General Discussion

A growing body of evidence from neuropsychological and normal studies supports the *obligatory phonological mediation hypothesis*. This study aimed to further investigate how phonological codes constrained the selection of orthographic codes in Chinese written production via the sublexical and lexical routes. In Experiment 1, we manipulated word frequencies of picture names and phonetic regularity of the first characters in a picture-naming task. Notably, writing latencies took longer for picture names with regular first characters than those with irregular ones in the low-frequency words, suggesting that phonological constraint on writing Chinese via the sublexical route. In Experiment 2, we employed a symbol—word associative written generation task to examine the HD effect in writing, and found that the sparse HD characters were written faster than the dense HD characters. HD is a phonological property at the lexical level, this finding therefore indicated that phonology constrained written production via the lexical route. Therefore, our findings demonstrated the presence of sublexical and lexical routes from phonology to orthography in Chinese written production.

### The phonetic regularity effect at the sublexical level

The direction of the phonetic regularity effect in the low-frequency condition was opposite to the typical facilitatory phonetic regularity effect found in reading [29–32, 53, 54, 60] and writing [33–35] tasks. In the framework of the dual-route written production model, Delattre et al. [33] proposed that lexical and sublexical routes operate in parallel to access orthographic codes from phonological codes. The lexical route retrieves spellings from a long-term memory storage of word-specific knowledge, and the sublexical route assembles spellings via a sublexical phoneme-to-grapheme conversion system. In the case of irregular words, the sublexical conversion route produced phonologically plausible errors, which was inconsistent with lexical orthographic codes. By contrast, the lexical and sublexical phonological codes were consistent for regular words. Therefore, writing an irregular character took longer than writing a regular character.

However, Snowling et al. [61] reported a case of developmental dyslexia in a patient (JM) who had severe difficulty in sublexical phonological coding, as revealed by his poor performance in naming nonwords, rhyme detection, and word/nonword repetition in spite of being able to recognize visual words. Snowling et al. examined JM’s word reading latencies as a function of WF and regularity, and found that JM took longer to read regular words than irregular words in the low WF condition. They suggested that slow but correct responses to regular words might be attributed to the retarded sublexical phonological route, which the subject could limitedly use, albeit slowly, to decode regular words. In other words, the sublexical route plays a role in reading regular words. If this route is damaged or cannot be efficiently used, the processing of regular words would be delayed more than that of irregular words.

Concerning the scenario in Chinese, some characteristics of Chinese characters might lead to the insufficient use of the sublexical route. As aforementioned, regular characters have similar pronunciations to their phonetic radicals. Given the multiple homophones for a syllable in Chinese, one pronunciation might connect to multiple phonetic radicals. For instance, the phonetic radicals of沥 (/li4/, to drip), 莉 (/li4/, jasmine), and 粒 (/li4/, grain) are历 (/li4/, calendar), 利 (/li4/, benefit), and 立(/li4/, to stand), respectively. All of these characters are phonetically regular, and their phonetic radicals are pronounced in the same pinyin. If one wants to write 沥, the sublexical route for retrieving the phonetic radical from the pronunciations should generate历. However, other homophonic phonetic radicals (i.e., “利” and “立”) are also activated and compete with the target phonetic radical. We postulated that this one-to-many mapping between phonology-to-orthography would make the sublexical route inefficient, and delayed the retrieval of phonetic radicals. By contrast, the phonetic radicals of irregular characters were accessed via the lexical route while bypassing the sublexical route. Hence, regular characters were written more slowly than irregular characters in Chinese, which resulted from the selection of corresponding orthographic codes. Sublexical sound—spelling mapping in alphabetical languages is much more transparent than that in Chinese, and the sublexical route is efficient and fast for regular words, which can lead to a facilitatory phonetic regularity effect.

The phonetic regularity effect emerged only in the low-frequency words because the fast lexical route plays a major role in the high-frequency words [21], regardless of regular or irregular words. By contrast, for the low-frequency words, the lexical and sublexical routes both play key roles in the low-frequency words; thus, the characteristic at the sublexical level affects the writing latencies. This speculation was consistent with findings of large regularity effects for low-frequency words in reading Chinese [29, 33, 53].

### The HD effect at the lexical level

In Experiment 2, we found an inhibitory HD effect, indicating that phonology constrained orthographic output in writing via the lexical route. There were multiple possible origins for the HD effect in Chinese written production. We attempted to account for this effect in the framework of speech and written production. Although spoken and written language production systems obviously share some processing levels, such as conceptual preparation and lexical selection, both have some specific processes [62]. They are thought to differ beyond the conceptual semantic level, namely, a phonological lexeme level for speaking and an orthographic lexeme level for writing [63–65]. Phonological information serves as input for articulation in spoken production, and orthographic information serves as input for orthographic output in written production. If written production does not depend on spoken production, the HD, as one of the lexical phonological properties, would not affect processes of written production. We observed a reliable HD effect, which reflected that the lexical phonological route mediated Chinese written production. In other words, written production was dependent on spoken production, and the HD effect originated from the phonological lexeme level.

Based on the written production model (see Fig 1), the HD effect possibly originated from the orthographic lexeme level as well. Bonin et al. [14]’s model proposed a semantic system that is symmetrically linked to both phonological and orthographic output lexicons in writing. Both lexicons also directly map onto each other, implying that selection of a graphemic entry is affected by indirect activation from the phonological lexicon. The cohort of homophone mates spread the activation from the phonological lexicon to the orthographic lexicon, and the competition in the dense HD group was stronger than that in the sparse HD group. Therefore, our results suggested that the lexical route was used for phonological mediation, especially when the sublexical characteristics, such as regularity and consistency, were matched between dense and sparse HD conditions.

Most studies manipulated the token syllable frequency and did not disassociate HD (or the type measure of syllable frequency) from the token syllable frequency. Studies demonstrated the facilitatory effects of the token frequency in spoken production (e.g., Spanish [66], Dutch [39, 40], French [67]), thereby providing evidence for the *mental syllabary* hypothesis(see [40] for details). In an interesting work by Conrad et al. [43], they independently manipulated type and token syllable frequency in a lexical decision task. Critically, they found a dissociation pattern between these two indexes. The token syllable frequency led to inhibitory effects at a lexical processing stage, whereas the type of syllable frequency led to facilitatory effects at a prelexical processing stage, reflecting the involvement of two different processes in the effects of syllable frequency. By contrast, Conrad et al. [68] observed an inhibitory effect of syllable frequency for words but a facilitatory effect for nonwords with a high-frequency first syllable in a German naming task.

However, the scenario is somewhat different in Chinese, a non-alphabetic language. In lexical decision and word-naming tasks, Ziegler et al. [38] manipulated the token syllable frequency. They reported that Chinese characters with a high syllable frequency are processed faster than those with a low syllable frequency when the characters were matched on visual character frequency and HD (the type syllable frequency). Unlike Ziegler et al., Chen et al. [44] varied HD while matching the token syllable frequency. They observed a facilitatory effect in the low visual frequency words in both lexical decision and word-naming tasks. By contrast, Wang et al. [45]observed an inhibitory effect of HD in an auditory lexical decision task (see [69] and [70] for similar findings). Zhang and Wang [27] observed a facilitatory effect of token syllable frequency in spoken and written picture-naming tasks; the magnitude of this effect in speaking was found to be larger than that in writing. According to these limited studies in Chinese, the token and type of syllable frequency might produce different influences on lexical decision and picture naming, which differed from general findings in alphabetic languages, such as Spanish, French, German, and Dutch.

Unlike previous studies that reported an exclusively facilitatory effect of token syllable frequency in *spoken* production, the current data suggested that a dense syllable cohort of monosyllabic words could be detrimental in *written* production. We interpreted the inhibitory HD effect as follows: the syllable of the target word activated all words that share this syllable in the mental lexicon. These co-activated candidates competed with the target word by lateral inhibition at the word unit level. Higher HD could result in a greater amount of lateral inhibition, so characters with higher HD would take longer to be lexically accessed (see also [45]).

To our knowledge, this paper is the first to report the effects of type syllable frequency in written production. Bonin et al. [14]’s Experiment1 did not find HD (1 vs. 2) effects in writing French picture names. This finding may be due to the small contrast in HD in their study. The inhibitory effect of token syllable frequency has been obtained in speech production as well. For example, Farrell and Abrams [71] reported an inhibitory effect of syllable frequency in successful speech production (see also [72] for inhibitory influences on tip-of-the-tongue resolution). Furthermore, they found that inhibitory syllable frequency effects were more pronounced in the presence of phonologically related distractors and for targets of low WF in PWI tasks. Their findings were consistent with lexical competition models. In particular, words with higher-frequency first syllables possess more syllabic neighbors and a greater number of high-frequency neighbors, and selecting a high-frequency syllable from a large cohort of activated lexical options in speech production takes longer time. Additionally, numerous studies have demonstrated the inhibitory effects of phonological neighborhood density in high-frequency words in spoken and written word recognition tasks [42, 73–75]. Our findings were consistent with findings in language production and visual word recognition. Considering the complicated situation in this issue, further investigations are necessary to clarify the different effects of token and type of syllable frequency.

Besides phonetic regularity and HD, WF was also manipulated in Experiments 1 and 2. The WF facilitatory effect was observed consistently in both experiments; picture names or words with high WF were produced faster than those with low WF. This finding was in line with previous findings on written production [27, 62, 76]. The absence of the interaction between WF and HD indicated that the two effects were independent, and they possibly affected different stages of written production (see [27] for a similar finding).

As mentioned in the introduction, a controversial issue in written production concerns the extent at which written production is dependent on phonology. We observed phonetic regularity effects and HD effects in writing Chinese, suggesting that the lexical and sublexical routes contributed to Chinese written production. This study provides evidence for the obligatory phonological mediation hypothesis. However, these results could not be regarded as evidence against the orthographic autonomy hypothesis, because this hypothesis did not deny the phonological role in written production. In line with this inference, some studies have shown evidence favoring the orthographic autonomy hypothesis [22, 23]. We suggested that a direct route from semantics to orthography and an indirect route from phonology to orthography (lexical and sublexical) were not exclusively incompatible with each other in the written production system.

In conclusion, we found a facilitatory effect of WF, in which pictures or words with high frequency were produced faster than those with low frequency. More importantly, we first reported the inhibitory effects of phonetic regularity and type syllable frequency (i.e., HD) in Chinese written production. Irregular characters were produced faster than regular ones in the low WF, and characters with sparse HD were written faster than those with dense HD. These findings suggested that phonological codes could constrain handwritten production via the lexical and sublexical routes.

## Supporting Information

S1 Appendix Stimuli used in Experiment 1. (DOCX)

S2 Appendix Stimuli used in Experiment 2. (DOCX)
