# Supplementary material for: Phonological Codes Constrain Output of Orthographic Codes via Sublexical and Lexical Routes in Chinese Written Production
Source: PLoS One. 2015 Apr 16;10(4):e0124470. doi: 10.1371/journal.pone.0124470 (PMC4400079; doi:10.1371/journal.pone.0124470)
Supplement: S1 Appendix — (DOCX) [file pone.0124470.s001.docx]

Appendix A: Stimuli used in Experiment 1.

| Condition | Picture Names | The Phonetic Radicals of The First Characters |
| --- | --- | --- |
| LF-Regular | 樱桃 (/ying1.tao2/, cherry) | 婴 (/ying1/, baby) |
|  | 铲车 (/chan3.che1/, bulldozer) | 产 (/chan3/, produce) |
|  | 菠萝 (/bo1.luo2/, pineapple) | 波 (/bo1/, wave) |
|  | 猩猩 (/xing1.xing1/, gorilla) | 星 (/xing1/, star) |
|  | 财宝 (/cai2.bao3/, treasure) | 才 (/cai2/, ability) |
|  | 轮胎 (/lun2.tai1/, tire) | 仑 (/lun2/, logical reasons) |
|  | 指纹 (/zhi3.wen2/, fingerprint) | 旨 (/zhi3/, aim) |
|  | 渔网 (/yu2.wang3/, fishing net) | 鱼 (/yu2/, fish) |
|  | 蚊子 (/wen2.zi5/, mosquito) | 文 (/wen2/, language) |
|  | 鳄鱼 (/e4.yu2/, crocodile) | 咢 (/e4/, drumming) |
|  | 球拍 (/qiu2.pai1/, racket) | 求 (/qiu2/, beg) |
|  | 锤子 (/chui2.zi5/, hammer) | 垂 (/chui2/, hang down) |
|  | 钩子 (/gou1.zi5/, hook) | 勾 (/gou1/, tick off) |
|  | 蚂蚁 (/ma3.yi3/, ant) | 马 (/ma3/, horse) |
|  |  |  |
| LF-Irregular | 笔头 (/bi3.tou2/, nib) | 毛 (/mao2/, hair) |
|  | 滑梯 (/hua2.ti1/, slide) | 骨 (/gu3/, bone) |
|  | 钳子 (/qian2.zi5/, pliers) | 甘 (/gan1/, sweet) |
|  | 凉鞋 (/liang2.xie2/, sandal) | 京 (/jing1/, capital) |
|  | 短裤 (/duan3.ku4/, shorts) | 豆 (/dou4/, bean) |
|  | 钻石 (/zuan4.shi2/, diamond) | 占 (/zhan4/, occupy) |
|  | 柜子 (/gui4.zi5/, cabinet) | 巨 (/ju4/, huge) |
|  | 佛像 (/fo2.xiang4/, buddha) | 弗 (/fu2/, not) |
|  | 秒表 (/miao3.biao3/, stop-watch) | 少 (/shao3/, few) |
|  | 棒球 (/bang4.qiu2/, baseball) | 奉 (/feng4/, abide) |
|  | 衬衫 (/chen4.shan1/, shirt) | 寸 (/cun4/, inch) |
|  | 蚌壳 (/bang4.ke2/, clam) | 丰 (/feng1/, abundant) |
|  | 烟斗 (/yan1.dou3/, pipe) | 因 (/yin1/, cause) |
|  | 硬币 (/ying4.bi4/, coin) | 更 (/geng4/, even more) |
|  |  |  |
| HF-Regular | 护士 (/hu4.shi5/, nurse) | 户 (/hu4/, door) |
|  | 蜘蛛 (/zhi1.zhu1/, spider) | 知 (/zhi1/, to know) |
|  | 钢琴 (/gang1.qin2/, piano) | 冈 (/gang1/, ridge) |
|  | 炸弹 (/zha4.dan4/, bomb) | 乍 (/zha4/, suddenly) |
|  | 苹果 (/ping2.guo3/, apple) | 平 (/ping2/, flat　) |
|  | 裤子 (/ku4.zi5/, pants) | 库 (/ku4/, warehouse) |
|  | 拇指 (/mu3.zhi3/, thumb) | 母 (/mu3/, mother) |
|  | 楼梯 (/lou2.ti1/, stairs) | 娄 (/lou2/, a family name) |
|  | 镜子 (/jing4.zi5/, mirror) | 竟 (/jing4/, complete) |
|  | 蚯蚓 (/qiu1.yin3/, earthworm) | 丘 (/qiu1/, hillock) |
|  | 橡皮 (/xiang4.pi2/, eraser) | 象 (/xiang4/, elephant) |
|  | 蝗虫 (/huang2.chong2/, locust) | 皇 (/huang2/, sovereign) |
|  | 帽子 (/mao4.zi5/, hat) | 冒 (/mao4/, to risk) |
|  | 棺材 (/guan1.cai5/, coffin) | 官 (/guan1/, officer) |
|  |  |  |
| HF-Irregular | 钥匙 (/yao4.shi5/, key) | 月 (/yue4/, moon) |
|  | 被子 (/bei4.zi5/, quilt) | 皮 (/pi2/, skin) |
|  | 标枪 (/biao1.qiang1/, javelin) | 示 (/shi4/, show) |
|  | 笛子 (/di2.zi5/, flute) | 由 (/you2/, reason) |
|  | 螺钉 (/luo2.ding1/, screw) | 累 (/lei4/, tired) |
|  | 蜡烛 (/la4.zhu2/, candle) | 昔 (/xi1/, past) |
|  | 灯泡 (/deng1.pao4/, lightbul) | 丁 (/ding1/, fourth) |
|  | 沙发 (/sha1.fa1/, sofa) | 少 (/shao3/, few) |
|  | 绵羊 (/mian2.yang2/, sheep) | 帛 (/bo2/, silk) |
|  | 波浪 (/bo1.lang4/, wave) | 皮 (/pi2/, skin) |
|  | 骆驼 (/luo4.tuo5/, camel) | 各 (/ge4/, each) |
|  | 辣椒 (/la4.jiao1/, pepper) | 束 (/shu4/, bunch) |
|  | 港口 (/gang3.kou3/, harbour) | 巷 (/xiang4/, alley) |
|  | 池塘 (/chi2.tang2/, pond) | 也 (/ye3/, also) |

Note: LF=Low Word Frequency; HF=High Word Frequency.
