# Supplementary material for: Phonological Codes Constrain Output of Orthographic Codes via Sublexical and Lexical Routes in Chinese Written Production
Source: PLoS One. 2015 Apr 16;10(4):e0124470. doi: 10.1371/journal.pone.0124470 (PMC4400079; doi:10.1371/journal.pone.0124470)
Supplement: S2 Appendix — (DOCX) [file pone.0124470.s002.docx]

Appendix D: Stimuli used in Experiment 2.

| Low-Dense | High-Dense | Low-Sparse | High-Sparse |
| --- | --- | --- | --- |
| 湃 (/pai4/, sound of waves) | 冰 (/bing1/, ice) | 惦 (/dian4/, think of) | 倍 (/bei4/, double) |
| 帕 (/pa4/, handkerchief) | 馆 (/guan3/, public building) | 鸽 (/ge1/, pigeon) | 府 (/fu3/, mansion) |
| 敛 (/lian3/, gather) | 麻 (/ma1/, numb) | 辜 (/gu1/, guilt) | 股 (/gu3/, part) |
| 蒜 (/suan4/, garlic) | 姐 (/jie3/, elder sister) | 狐 (/hu2/, fox) | 湖 (/hu2/, lake) |
| 衅 (/xin4/, quarrel) | 航 (/hang2/, sail) | 俭 (/jian3/, frugal) | 减 (/jian3/, minus) |
| 腕 (/wan4/, wrist) | 抬 (/tai2/, uplift) | 椒 (/jiao1/, pepper) | 角 (/jiao3/, horn) |
| 佣 (/yong4/, servant) | 伤 (/shang1/, wound) | 绞 (/jiao3/, wring) | 境 (/jing4/, border) |
| 颖 (/ying3/, smart) | 护 (/hu4/, protect) | 梨 (/li2/, pear) | 颗 (/ke1/, grain) |
| 苔 (/tai2/, moss) | 供 (/gong4/, offer) | 栖 (/qi1/, roost) | 留 (/liu2/, reserve) |
| 沼 (/zhao3/, pool) | 刺 (/ci4/, sting) | 榕 (/rong2/, ficus) | 输 (/shu1/, lose) |
| 桨 (/jiang3/, oar) | 硬 (/ying4/, hard) | 枢 (/shu1/, pivot) | 尾 (/wei3/, tail) |
| 铛 (/dang1/, frying pan) | 罪 (/zui4/, crime) | 恕 (/shu4/, forgive) | 味 (/wei4/, taste) |
| 姥 (/lao3/, clank) | 显 (/xian3/, show) | 蔚 (/wei4/, luxuriant) | 歌 (/ge1/, song) |
| 嘀 (/di1/, murmur) | 剩 (/sheng4/, surplus) | 萧 (/xiao1/, sorrowful) | 协 (/xie2/, harmonize) |
| 魄 (/po4/, soul) | 罢 (/ba4/, cease) | 谐 (/xie2/, harmonious) | 续 (/xu4/, continuous) |
| 妆 (/zhuang1/, adornment) | 掉 (/diao4/, drop) | 絮 (/xu4/, raw cotton) | 沿 (/yan2/, along) |
| 涩 (/se4/, unsmooth) | 祖 (/zu3/, ancestor) | 雁 (/yan4/, goose) | 移 (/yi2/, shift) |
| 脓 (/nong2/, purulency) | 侵 (/qin1/, intrude) | 荧 (/ying2/, fluorescent) | 英 (/ying1/, hero) |
| 钦 (/qin1/, admire) | 摆 (/bai3/, pendulum) | 羽 (/yu3/, feather) | 语 (/yu3/, speech) |
| 芳 (/fang1/, fragrant) | 编 (/bian1/, compile) | 贞 (/zhen1/, chaste) | 针 (/zhen1/, needle) |

Note: Low=Low Frequency; High=High Frequency; Dense=Dense Homophone Density; Sparse=Sparse Homophone Density.
